# Association of Flavored Tobacco Use With Tobacco Initiation and Subsequent Use Among US Youth and Adults, 2013-2015

**DOI:** 10.1001/jamanetworkopen.2019.13804

**Published:** 2019-10-23

**Authors:** Andrea C. Villanti, Amanda L. Johnson, Allison M. Glasser, Shyanika W. Rose, Bridget K. Ambrose, Kevin P. Conway, K. Michael Cummings, Cassandra A. Stanton, Kathryn C. Edwards, Cristine D. Delnevo, Olivia A. Wackowski, Shari P. Feirman, Maansi Bansal-Travers, Jennifer K. Bernat, Enver Holder-Hayes, Victoria R. Green, Marushka L. Silveira, Andrew Hyland

**Affiliations:** 1 The Schroeder Institute for Tobacco Research and Policy Studies at Truth Initiative, Washington, DC; 2 Vermont Center on Behavior and Health, Department of Psychiatry, The University of Vermont, Burlington; 3 Office of Science, Center for Tobacco Products, Food and Drug Administration, Silver Spring, Maryland; 4 Division of Epidemiology, Services, and Prevention Research, National Institute on Drug Abuse, Bethesda, Maryland; 5 Department of Psychiatry & Behavioral Sciences, Medical University of South Carolina, Charleston; 6 Westat, Rockville, Maryland; 7 Department of Oncology, Lombardi Comprehensive Cancer Center, Georgetown University Medical Center, Washington, DC; 8 Department of Health Education and Behavioral Science, Center for Tobacco Studies, Rutgers School of Public Health, Piscataway, New Jersey; 9 Department of Health Behavior, Division of Cancer Prevention & Population Sciences, Roswell Park Cancer Institute, Buffalo, New York; 10 Kelly Government Solutions, Rockville, Maryland

## Abstract

**Importance:**

Flavors in tobacco products may appeal to young and inexperienced users.

**Objective:**

To examine among youth (aged 12-17 years), young adults (aged 18-24 years), and adults (aged ≥25 years) the prevalence of first use of flavored tobacco products among new tobacco users and the association between first flavored use of a given tobacco product and tobacco use 1 year later, including progression of tobacco use.

**Design, Setting, and Participants:**

This cohort study represents a longitudinal analysis of data from the Population Assessment of Tobacco and Health (PATH) Study, a nationally representative study with data collected in 2013 to 2014 (wave 1) and 2014 to 2015 (wave 2). Participants were noninstitutionalized individuals, including 11 996 youth and 26 447 adults, in selected households who participated in both waves of the PATH Study. Data analysis was conducted from July 2016 to June 2019.

**Main Outcomes and Measures:**

Prevalence of tobacco product use at wave 2.

**Results:**

The mean (SE) age of the participants was 14.5 (0.0) years for youth, 21.1 (0.0) years for young adults, and 50.3 (0.0) for adults. Most youth (71.9%; 95% CI, 69.7%-74.0%) and young adults (57.6%; 95% CI, 54.9%-60.3%) who were new users of tobacco products over the 10- to 13-month follow-up period used flavored products. First use of a menthol or mint or other flavored cigarette documented at wave 1 was positively associated with past 12-month and past 30-day cigarette use in all age groups at wave 2 compared with first use of a nonflavored cigarette (youth, flavored cigarette, past 12-month use adjusted prevalence ratio [aPR], 1.14 [95% CI, 1.05-1.25] and past 30-day use aPR, 1.15 [95% CI, 1.00-1.31]; youth, menthol or mint cigarette, past 12-month use aPR, 1.18 [95% CI, 1.08-1.29] and past 30-day use aPR, 1.19 [95% CI, 1.04-1.37]; young adult, flavored cigarette, past 12-month use aPR, 1.09 [95% CI, 1.04-1.15] and past 30-day use aPR, 1.13 [95% CI, 1.06-1.21]; young adult menthol or mint cigarette, past 12-month use aPR, 1.10 [95% CI, 1.05-1.16] and past 30-day use aPR, 1.15 [95% CI, 1.07-1.23]; adult flavored cigarette, past 12-month use aPR, 1.10 [95% CI, 1.05-1.15] and past 30-day use aPR, 1.09 [95% CI, 1.04-1.14]; adult menthol or mint cigarette, past 12-month use aPR, 1.13 [95% CI, 1.08-1.18] and past 30-day use aPR, 1.12 [95% CI, 1.07-1.17]). Among young adults, first use of flavored e-cigarettes (aPR, 2.05; 95% CI, 1.61-2.61), any cigars (aPR, 1.60; 95% CI, 1.26-2.02), cigarillos (aPR, 1.49; 95% CI, 1.08-2.05), filtered cigars (aPR, 3.69; 95% CI, 2.08-6.57), hookah (aPR, 1.91; 95% CI, 1.23-2.98), and any smokeless tobacco (aPR, 1.54; 95% CI, 1.08-2.20) was prospectively associated with current regular use of those products at wave 2 compared with first nonflavored use. Among adults aged 25 years and older, first use of flavored e-cigarettes (aPR, 1.60; 95% CI, 1.41-1.82), any cigars (aPR, 1.56; 95% CI, 1.29-1.87), cigarillos (aPR, 1.29; 95% CI, 1.01-1.64), filtered cigars (aPR, 1.79; 95% CI, 1.25-2.54), hookah (aPR, 5.66; 95% CI, 2.04-15.71), and any smokeless tobacco (aPR, 1.55; 95% CI, 1.32-1.82) was prospectively associated with current regular use of those products at wave 2 compared with first nonflavored use.

**Conclusions and Relevance:**

In this longitudinal cohort study, flavors in tobacco products were associated with youth and young adult tobacco experimentation. First use of a flavored tobacco product may place youth, young adults, and adults at risk of subsequent tobacco use.

## Key Points

**Question:**

What is the association between first flavored use of a given tobacco product and subsequent tobacco use, including progression of tobacco use, among US youth (aged 12-17 years), young adults (aged 18-24 years), and adults (aged ≥25 years)?

**Findings:**

In this cohort study of 11 996 youth and 26 447 adults who participated in waves 1 and 2 of the Population Assessment of Tobacco and Health Study, most youth and young adult new tobacco users first tried a flavored product. First use of flavored tobacco products was positively associated with subsequent product use compared with first use of a nonflavored product.

**Meaning:**

First use of flavored tobacco products may place youth and adults at risk of subsequent tobacco use.

## Teaser

This cohort study uses data from the Population Assessment of Tobacco and Health (PATH) Study to examine whether first use of flavored tobacco products is associated with subsequent tobacco use among US youth and adults.

## Introduction

Children prefer sweet flavors more than adults do,^1^ and tobacco industry documents^2,3,4,5^ confirm that flavors in tobacco products can increase their appeal to young and inexperienced tobacco users. Consistent with studies^6,7,8^ on menthol cigarettes and flavored cigars, data from the first wave of the Population Assessment of Tobacco and Health (PATH) Study^9,10,11^ revealed a strong inverse age gradient in the prevalence of flavored tobacco product use, with the highest use among youth aged 12 to 17 years, followed by young adults aged 18 to 24 years, and the lowest use among adults aged 25 years and older. These data^9,10^ also show a strong association between first use of a flavored tobacco product and current tobacco use among youth and adults.

Few longitudinal studies to date have examined the association between flavored tobacco product use and initiation or continuation of tobacco use, and these studies^12,13,14^ have largely been limited to menthol cigarettes. These studies highlight that menthol brand recognition is associated with smoking experimentation among youth,^12^ that adolescents who initiate smoking with menthol cigarettes are more likely to progress to established smoking by the end of 3 years than those who initiated with nonmenthol cigarettes,^13^ and that prior initiation with a menthol cigarette compared with a nonmenthol cigarette is associated with current cigarette smoking at follow-up among young adults.^14^ Five other cross-sectional studies^10,15,16,17,18^ support these findings.

The current study extends prior research by leveraging longitudinal data from waves 1 and 2 of the PATH Study to assess whether there is a prospective association between first flavored use of a given tobacco product and subsequent use of that specific product (eg, e-cigarettes). In addition, this study examines whether first use of a flavored tobacco product at wave 1 is associated with progression to greater frequency of tobacco use at wave 2. The primary aims of this study are to report the proportions of new tobacco users at wave 2 whose first use of a given tobacco product was flavored (ie, first flavored use) and to assess the association between first flavored use of a given tobacco product at wave 1 and subsequent tobacco use, including frequency of tobacco use, at wave 2 for youth (aged 12-17 years), young adults (aged 18-24 years), and adults (aged ≥25 years).

## Methods

The National Institutes of Health, through the National Institute on Drug Abuse, is partnering with the US Food and Drug Administration’s Center for Tobacco Products to conduct the PATH Study under a contract with Westat. The PATH Study is an ongoing, nationally representative, longitudinal cohort study of adults and youth in the United States. The PATH Study uses audio computer-assisted self-interviews available in English and Spanish to collect self-reported information on tobacco-use patterns and associated health behaviors. Wave 1 data collection was conducted from September 12, 2013, to December 14, 2014; wave 2 data were collected from October 23, 2014, to October 30, 2015. The PATH Study recruitment used a stratified, address-based, area-probability sampling design at wave 1 that oversampled adult tobacco users, young adults (aged 18-24 years), and African American adults. An in-person screener was used at wave 1 to select youth and adults from households for participation.

The PATH study was conducted by Westat and approved by the Westat institutional review board. All participants aged 18 years and older provided written informed consent, with youth participants aged 12 to 17 years providing assent while their parent or legal guardian provided written informed consent. This study follows the Strengthening the Reporting of Observational Studies in Epidemiology (STROBE) reporting guideline for observational studies.^19^

Population and replicate weights using the balanced repeated replication method with the Fay adjustment (ρ = 0.3) were created that adjusted for the complex study design characteristics (eg, oversampling at wave 1) and nonresponse at waves 1 and 2. Combined with the use of a probability sample, the weights allow analyses of the PATH Study data to compute estimates that are robust and representative of the noninstitutionalized, civilian US population aged 12 years and older. The longitudinal sampling weights provided for wave 2 are adjusted for wave 2 nonresponse to ensure that the wave 1 sample is representative of the population in the longitudinal estimates. Further details regarding the PATH Study design and methods have been published elsewhere.^20^ Details on survey interview procedures, questionnaires, sampling, weighting, and information on accessing the data are available online.^21^

At wave 1, the weighted response rate for the household screener was 54.0%. Among households that were screened, the overall weighted response rate at wave 1 was 74.0% for the adult interview and 78.4% for the youth interview. At wave 2, the overall weighted response rate was 83.2% for the adult interview and 87.3% for the youth interview.

At wave 1, interviews were completed with 32 320 adults (aged ≥18 years) and 13 651 youth (aged 12-17 years). At wave 2, interviews were completed with 28 362 adults and 12 172 youth. The differences in number of completed interviews between wave 1 and wave 2 reflect attrition due to nonresponse, mortality, and other factors. The sample at wave 2 also includes 1915 youth aged 17 years at wave 1 who responded to the youth questionnaire in wave 1 and then turned 18 and responded to the adult questionnaire in wave 2.^21^ Between waves 1 and 2, retention rates were 88.4% for continuing youth, 83.1% for continuing adults, and 85.7% for aged-up adults (18-year-olds at wave 2). Analyses in this study focus on respondents who provided data for both study waves (11 996 youth and 26 447 adults); unless otherwise stated, any references to age are based on age at wave 1.

### Measures

#### Tobacco Product Use

Ever and current tobacco use was assessed at waves 1 and 2 among youth, young adults, and adults for cigarettes, e-cigarettes, traditional cigars, cigarillos, filtered cigars, hookah tobacco, pipe tobacco, smokeless tobacco (eg, moist snuff or chew), snus pouches, and dissolvable tobacco. Any cigar use was defined as using traditional cigars, cigarillos, or filtered cigars. Any smokeless tobacco use was defined as using smokeless tobacco or snus pouches. Youth, young adults, and adults who tried a tobacco product for the first time between waves 1 and 2 were defined as new users, with age at tobacco trial defined as their age at wave 1. Current use was defined in multiple ways as outlined in previous analyses^22^ and in the eTable in the Supplement. Participants missing data on moderate, frequent, or daily use of a product because of an instrument skip pattern were coded as not having the outcome and were included in the denominator.

#### Wave 1 First Flavored Tobacco Product Use

At wave 1, ever cigarette users were asked whether, when they first used a cigarette (youth) or when they first started smoking cigarettes (adults), it was “flavored to taste like menthol or mint.” Ever cigarette users who replied “no” to the menthol question were then asked whether their first cigarette was flavored to taste like “clove, spice, candy, fruit, chocolate, alcohol (such as wine or cognac), or other sweets.” Two comparisons were made for cigarettes: first use of any flavored cigarette (including menthol/mint, as indicated in the survey) vs first use of a nonflavored cigarette, and first use of a menthol or mint flavored cigarette vs first use of a nonflavored cigarette with individuals who reported other first flavored cigarette use excluded from the denominator. Ever users of other tobacco products were queried about whether, when they first used the product (youth) or when they first started using the product (adults), it was “flavored to taste like menthol, mint, clove, spice, candy, fruit, chocolate, alcohol (such as wine or cognac), or other sweets” (eTable in the Supplement).

#### Wave 1 Covariates

All covariates were assessed at wave 1 and were selected on the basis of previous work^10^ using this data set (eTable in the Supplement). Sociodemographic variables included self-reported age (adult only), sex, race/ethnicity, educational attainment, and annual household income (adults only). Past 30-day use of alcohol, marijuana, and other drugs (eg, painkillers, sedatives, tranquilizers, or stimulants) was assessed; never users were categorized as reporting no past 30-day use. Respondents also completed the Global Appraisal of Individual Needs–Short Screener,^23^ which measures severity of symptoms on 3 subscales (internalizing problems, externalizing problems, and substance use problems) in the past year (ie, 0-1 symptoms [no or low], 2-3 symptoms [moderate], and 4 or ≥4 symptoms [high], depending on the scale).

### Statistical Analysis

Data analysis was conducted from July 2016 to June 2019. Analyses were conducted using svy procedures in Stata/SE statistical software version 14.2 (StataCorp) to account for the complex study design. Analysis of new users focused on the prevalence of using a flavored product at first tobacco use at wave 2 (Figure). For all other analyses, the main outcome was current product-specific use at wave 2 as defined in the eTable in the Supplement. The prevalence of each outcome was estimated for youth, young adults, and adults aged 25 years and older according to age at wave 1. Estimates with denominators less than 50 or relative SE greater than 30% were suppressed.^24^ Missing data on age, sex, race or Hispanic ethnicity, and adult education were imputed at wave 1 as described elsewhere.^21^ Participants missing any response to a composite variable (eg, any past 30-day tobacco use) were treated as missing; missing data were handled with listwise deletion. Multivariable models were built separately for youth, young adults, and adults aged 25 years or older; all models included sex, race/ethnicity, education, past 30-day alcohol, marijuana, or other drug use, and the 3 Global Appraisal of Individual Needs–Short Screener subscales as covariates. Adult models also included age and income. Modified Poisson regression models^25^ estimated the association between first flavored tobacco use among ever users at wave 1 and current tobacco use at wave 2, as well as moderate, frequent, and daily use at wave 2. For the 3 products for which there were sufficient sample sizes in each of the age groups (flavored cigarettes, menthol cigarettes, and flavored e-cigarettes), we conducted multivariable multinomial logistic regression models of increasing frequency of tobacco use compared with no use from the mutually exclusive categories of tobacco use frequency. For all analyses, α was set at *P* < .05 using 2-sided tests. Stata’s svy commands used a logit transformation to produce confidence intervals with limits between 0 and 1.^26^

**Figure.**
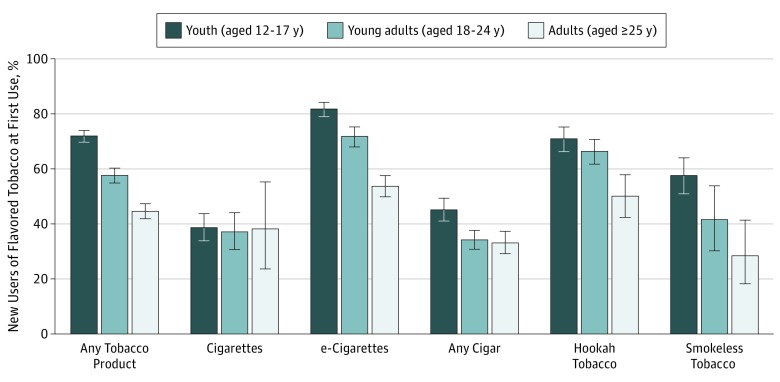
Weighted Proportions of New Tobacco Users at Wave 2 Who Reported Using a Flavored Product at First Use Percentages are weighted to represent the US population, and 95% CIs (whiskers) are estimated using the balanced repeated replication method. New use is ascribed to the participants’ age at wave 1. Respondents were categorized into age groups (youth aged 12-17 years, young adults aged 18-24 years, and adults aged ≥25 years) according to their ages at wave 1. New use of a tobacco product is defined as starting to use a product between waves 1 and 2. This can include never users at wave 1 who start tobacco use at wave 2 and ever users at wave 1 who report use of a new product or products at wave 2. Individuals who reported “don’t know” or refused to answer any part of the definition of ever use or first flavored use were excluded from the denominator. Unweighted numbers and unweighted percentages are presented for each age group: Among 11 996 youth, 2136 (17.8%) reported new use of a tobacco product, 9622 (80.2%) reported no new initiation, and 238 (2.0%) did not provide information on initiation between wave 1 and wave 2. Among 7325 young adults, 2058 (24.9%) reported new use of a tobacco product, 5232 (74.7%) reported no new initiation, and 35 (0.4%) did not provide information on initiation between wave 1 and wave 2. Among 19 116 adults aged 25 years and older, 2580 (8.1%) reported new use of a tobacco product, 16 407 (91.4%) reported no new initiation, and 129 (0.5%) did not provide information on initiation between wave 1 and wave 2. First flavored use is defined as reporting that the first product used was “flavored to taste like menthol, mint, clove, spice, candy, fruit, chocolate, alcohol (such as wine or cognac), or other sweets.” Individuals who did not report “yes,” “no,” or “I don’t know” or refused to answer whether their first product was flavored were excluded from the denominator. Flavored pipe tobacco and dissolvable tobacco use was not assessed among youth. Unweighted numbers and unweighted percentages are presented for each age group. For 2136 youth new tobacco users, 95 (4.5%) did not report whether they had used any flavored product between wave 1 and wave 2. For 2058 young adult new tobacco users, 58 (2.8%) did not report whether they had used any flavored product between wave 1 and wave 2. For 2580 adult (aged ≥25 years) new tobacco users, 58 (2.3%) did not report whether they had used any flavored product between wave 1 and wave 2. Any tobacco product included cigarettes, e-cigarettes, traditional cigars, cigarillos, filtered cigars, hookah, pipe (for adults only), smokeless tobacco, and snus or dissolvable tobacco (for adults only); any cigar use reflects use of a traditional cigar, cigarillo, or filtered cigar. Data are from the Population Assessment of Tobacco and Health (PATH) Study,^9,10,11^ waves 1 and 2.

## Results

The mean (SE) age of the participants at wave 2 was 14.5 (0.0) years for youth, 21.1 (0.0) years for young adults, and 50.3 (0.0) for adults. For adults who completed interviews at both waves, the comparable weighted age distributions were 13.0% aged 18 to 24 years, 8.5% aged 25 to 29 years, 9.1% aged 30 to 34 years, 16.7% aged 35 to 44 years, 34.6% aged 45 to 64 years, and 18.0% aged 65 years and older at wave 1. Detailed characteristics of the adult sample are presented elsewhere.^27^

### Prevalence of First Use Being a Flavored Tobacco Product, Among New Users at Wave 2

Between waves 1 and 2, 12.1% of youth (aged 12-17 years), 27.6% of young adults (aged 18-24 years), and 8.3% of adults (aged ≥25 years) became new users of a tobacco product (ie, were never users of a given product at wave 1; weighted percentages). Of these, first use of any product being flavored was inversely associated with age, with 71.9% (95% CI, 69.7%-74.0%) of youth, 57.6% (95% CI, 54.9%-60.3%) of young adults, and 44.6% (95% CI, 41.8%-47.4%) of adults aged 25 years and older whose first use of any tobacco product was flavored (Figure). First use of e-cigarettes and hookah being flavored was more prevalent among youth (e-cigarettes, 81.7% [95% CI, 78.9%-84.2%]; hookah, 70.9% [95% CI, 66.2%-75.2%]) and young adults (e-cigarettes, 71.7% [95% CI, 68.0%-75.2%]; hookah, 66.4% [95% CI, 61.8%-70.7%]) compared with adults aged 25 years and older (e-cigarettes, 53.7% [95% CI, 49.9%-57.5%]; hookah, 50.1% [95% CI, 42.3%-57.8%]); first use of any cigar and smokeless tobacco being flavored was more prevalent among youth (any cigar, 45.2% [95% CI, 41.1%-49.3%]; smokeless tobacco, 57.6% [95% CI, 50.9%-64.0%]) compared with adults aged 25 years and older (any cigar, 33.0% [95% CI, 29.1%-37.2%]; smokeless tobacco, 28.4% [95% CI, 18.2%-41.3%]).

### Association Between First Use of a Given Tobacco Product Being Flavored at Wave 1 and Current Tobacco Use at Wave 2

Among ever tobacco users at wave 1, first use of a flavored cigarette was positively associated with past 12-month and past 30-day cigarette use, either flavored or unflavored, at wave 2 in all 3 age groups (Tables 1, 2, and 3) compared with first use of nonflavored cigarette, after adjusting for covariates (youth, past 12-month use adjusted prevalence ratio [aPR], 1.14 [95% CI, 1.05-1.25] and past 30-day use aPR, 1.15 [95% CI, 1.00-1.31]; young adult, past 12-month use aPR, 1.09 [95% CI, 1.04-1.15] and past 30-day use aPR, 1.13 [95% CI, 1.06-1.21]; adult, past 12-month use aPR, 1.10 [95% CI, 1.05-1.15] and past 30-day use aPR, 1.09 [95% CI, 1.04-1.14]). First use of a menthol or mint flavored cigarette among ever cigarette users at wave 1 was also positively associated with past 12-month and past 30-day cigarette use among youth (past 12-month use aPR, 1.18 [95% CI, 1.08-1.29] and past 30-day use aPR, 1.19 [95% CI, 1.04-1.37]), young adults (past 12-month use aPR, 1.10 [95% CI, 1.05-1.16] and past 30-day use aPR, 1.15 [95% CI, 1.07-1.23]), and adults aged 25 years and older (past 12-month use aPR, 1.13 [95% CI, 1.08-1.18] and past 30-day use aPR, 1.12 [95% CI, 1.07-1.17]) at wave 2 compared with first use of a nonflavored cigarette. First use of any flavored smokeless product was also prospectively associated with past 30-day any smokeless product use among youth aged 12 to 17 years (aPR, 1.76; 95% CI, 1.21-2.57) (Table 1). Low sample sizes limited estimation of the association between first use of a flavored tobacco product and moderate, frequent, daily, and current regular use of that product among youth (Table 1).

**Table 1.** Association Between First Tobacco Product Flavored Among Youth Ever Tobacco Users at Wave 1 and Product-Specific Tobacco Use at Wave 2 of the Population Assessment of Tobacco and Health Study

Ever Tobacco Use at Wave 1	Past 12-mo Use at Wave 2^a^		Past 30-d Use at Wave 2^b^		≥6 d Use at Wave 2^c^		≥20 d Use at Wave 2^c^		Daily Product Use at Wave 2^c^	
	Participants, No. (%)	aPR (95% CI)^d^	Participants, No. (%)	aPR (95% CI)^d^	Participants, No. (%)	aPR (95% CI)^d^	Participants, No. (%)	aPR (95% CI)^d^	Participants, No. (%)	aPR (95% CI)^d^
Ever cigarette use										
First product nonflavored	453 (60)	1 [Reference]	293 (39)	1 [Reference]	178 (23.9)	1 [Reference]	130 (17.2)	1 [Reference]	99 (12.8)	1 [Reference]
First product flavored	531 (68.3)	1.14 (1.05-1.25)	342 (44.94)	1.15 (1.00-1.31)	193 (24.9)	1.05 (0.85-1.29)	142 (17.8)	1.11 (0.85-1.47)	107 (13.4)	1.21 (0.92-1.59)
First product menthol or mint flavored^e^	470 (69.9)	1.18 (1.08-1.29)	309 (46.8)	1.19 (1.04-1.37)	174 (25.9)	1.09 (0.88-1.34)	129 (18.6)	1.17 (0.88-1.56)	99 (14.2)	1.30 (0.97-1.72)
Ever e-cigarette use										
First product nonflavored	122 (52.5)	1 [Reference]	58 (23.3)	1 [Reference]	22 (9)	1 [Reference]	NA^f^	1 [Reference]	NA^f^	1 [Reference]
First product flavored	569 (58.9)	1.12 (0.96-1.29)	266 (28.4)	1.19 (0.91-1.55)	105 (11.8)	1.39 (0.82-2.36)	55 (6.2)	1.67 (0.77-3.63)	42 (4.5)	2.29 (0.81-6.50)
Ever any cigar use										
First product nonflavored	182 (64.9)	1 [Reference]	97 (36.3)	1 [Reference]	NA^f^	1 [Reference]	NA^f^	1 [Reference]	NA^f^	NA^f^
First product flavored	398 (69.8)	1.08 (0.96-1.21)	182 (33.7)	0.94 (0.72-1.21)	35 (6.5)	NA^g^	18 (3.4)	NA^g^	NA^f^	NA^f^
Ever use of traditional cigar										
First product nonflavored	85 (65)	1 [Reference]	41 (31.3)	1 [Reference]	NA^f^	NA^f^	NA^f^	NA^f^	NA^f^	NA^f^
First product flavored	88 (69.4)	1.16 (0.98-1.37)	34 (27.6)	0.92 (0.57-1.49)	NA^f^	NA^f^	NA^f^	NA^f^	NA^f^	NA^f^
Ever use of cigarillos										
First product nonflavored	160 (64.3)	1 [Reference]	83 (34.8)	1 [Reference]	NA^f^	1 [Reference]	NA^f^	NA^f^	NA^f^	NA^f^
First product flavored	307 (63.4)	1.00 (0.87-1.15)	128 (27.7)	0.81 (0.61-1.07)	17 (3.7)	NA^g^	NA^f^	NA^f^	NA^f^	NA^f^
Ever use of filtered cigars										
First product nonflavored	51 (57.3)	1 [Reference]	13 (14.4)	1 [Reference]	NA^f^	NA^f^	NA^f^	NA^f^	NA^f^	NA^f^
First product flavored	118 (65)	1.10 (0.84-1.44)	29 (14.8)	0.95 (0.39-2.34)	NA^f^	NA^f^	NA^f^	NA^f^	NA^f^	NA^f^
Ever use of pipe tobacco										
First product nonflavored	53 (35.9)	1 [Reference]	17 (11.9)	NA^f^	NA^f^	NA^f^	NA^f^	NA^f^	NA^f^	NA^f^
First product flavored	21 (31.2)	0.77 (0.41-1.46)	NA^f^	NA^f^	NA^f^	NA^f^	NA^f^	NA^f^	NA^f^	NA^f^
Ever use of hookah tobacco										
First product nonflavored	56 (56)	1 [Reference]	23 (24.8)	1 [Reference]	NA^f^	1 [Reference]	NA^f^	1 [Reference]	NA^f^	NA^f^
First product flavored	480 (64)	1.09 (0.87-1.37)	183 (24.5)	0.91 (0.55-1.51)	38 (4.8)	0.62 (0.23-1.67)	19 (2.5)	0.62 (0.16-2.46)	NA^f^	NA^f^
Ever use of any smokeless tobacco^h^										
First product nonflavored	51 (41.6)	1 [Reference]	24 (19.5)	1 [Reference]	13 (11)	1 [Reference]	NA^f^	1 [Reference]	NA^f^	1 [Reference]
First product flavored	216 (54.5)	1.29 (0.99-1.68)	132 (33.9)	1.76 (1.21-2.57)	77 (20.2)	1.77 (0.92-3.40)	65 (17.1)	1.94 (0.92-4.12)	49 (12.5)	NA^g^
Ever use of smokeless tobacco (excluding snus)										
First product nonflavored	65 (48.1)	1 [Reference]	34 (25.1)	1 [Reference]	23 (17.7)	1 [Reference]	21 (16.3)	1 [Reference]	18 (14)	1 [Reference]
First product flavored	169 (51.3)	1.12 (0.88-1.41)	106 (32.6)	1.32 (0.98-1.79)	63 (19.7)	1.05 (0.67-1.65)	54 (16.6)	0.98 (0.61-1.59)	40 (12.2)	NA^g^
Ever use of snus										
First product nonflavored	NA^f^	1 [Reference]	NA^f^	1 [Reference]	NA^f^	NA^f^	NA^f^	NA^f^	NA^f^	NA^f^
First product flavored	80 (50.1)	1.37 (0.67-2.80)	32 (19.7)	1.22 (0.26-5.60)	NA^f^	NA^f^	NA^f^	NA^f^	NA^f^	NA^f^
Ever use of dissolvable tobacco										
First product nonflavored	NA^f^	NA^f^	NA^f^	NA^f^	NA^f^	NA^f^	NA^f^	NA^f^	NA^f^	NA^f^
First product flavored	NA^f^	NA^f^	NA^f^	NA^f^	NA^f^	NA^f^	NA^f^	NA^f^	NA^f^	NA^f^

Abbreviations: aPR, adjusted prevalence ratio; NA, not applicable.

^a^ Past 12-month use was defined as smoked or used product (even 1 or 2 times) in the past 12 months. Individuals who responded “don’t know” or refused to answer were excluded from the denominator. Unweighted numbers (percentages) of respondents excluded from the denominator for past 12-month use were as follows: cigarettes, 0 (0.0%); e-cigarettes, 26 (2.1%); any cigar, 2 (0.2%); traditional cigars, 1 (0.4%); cigarillos, 2 (0.3%); filtered cigars, 1 (0.4%); pipe, 2 (0.9%); hookah, 1 (0.1%); any smokeless product, 6 (1.1%); smokeless product, 4 (0.8%); and snus, 2 (1.0%).

^b^ Past 30-day use was defined as smoked or used product (even 1 or 2 times) in the past 30 days. Individuals who responded “don’t know” or refused to answer were excluded from the denominator. Unweighted numbers (percentages) of respondents excluded from the denominator for past 30-day use were as follows: cigarettes, 3 (0.2%); e-cigarettes, 27 (2.2%); any cigar, 34 (3.9%); traditional cigars, 2 (0.8%); cigarillos, 31 (4.2%); filtered cigars, 1 (0.4%); pipe, 2 (0.9%); hookah, 4 (0.5%); any smokeless product, 8 (1.5%); smokeless product, 5 (1.0%); and snus, 1 (0.5%).

^c^ Moderate use was defined as having smoked or used the product on at least 6 of the past 30 days. Frequent product use was defined as having smoked or used the product on at least 20 of the past 30 days. Daily use among youth was defined as having smoked or used the product on 30 of the past 30 days. Individuals who responded “don’t know” or refused to answer were excluded from the denominator. Unweighted numbers (percentages) of respondents excluded from the denominator for moderate, frequent, and daily use were as follows: cigarettes moderate or frequent use, 25 (1.6%); cigarettes daily use, 12 (0.8%); e-cigarettes moderate or frequent use, 60 (4.9%); e-cigarettes daily use, 34 (2.8%); any cigar product moderate use, 54 (6.3%); any cigar product frequent use, 57 (6.7%); cigarillos moderate use, 29 (3.9%); hookah moderate or frequent use, 11 (1.3%); any smokeless tobacco product moderate use, 18 (3.4%); any smokeless tobacco product frequent use, 19 (3.5%); any smokeless tobacco product daily use, 10 (1.9%); smokeless tobacco moderate or frequent use, 11 (2.3%); and smokeless tobacco daily use, 7 (1.5%).

^d^ Multivariable modified Poisson regression models among youth were adjusted for sex, race/ethnicity, education, past 30-day alcohol, marijuana, or other drug use, and 3 Global Appraisal of Individual Needs–Short Screener subscales (internalizing problems, externalizing problems, and substance use problems). Individuals who reported “don’t know” or refused to answer any of these items were treated as missing. Among youth ever tobacco users, data were missing for sex (0 participants [0.0%]), race/ethnicity (0 participants [0.0%]), education (128 participants [5.2%]), past 30-day alcohol, marijuana, or other drug use (64 participants [2.6%]), internalizing problems subscale (52 participants [2.1%]), externalizing problems subscale (93 participants [3.8%]), and substance use problems subscale (91 participants [3.7%]). Respondents who reported never alcohol, marijuana, or other drug use were categorized as non–past 30-day users.

^e^ A separate comparison was made between wave 1 cigarette smokers who first smoked a menthol or mint flavored cigarette vs smokers who first smoked a nonflavored cigarette. Individuals who reported first other flavored cigarette use were excluded from the denominator.

^f^ The estimate was suppressed because it has low statistical precision. It is based on a sample size of less than 50, or the coefficient of variation of the estimate is larger than 30%.

^g^ There were insufficient observations to compute balanced repeated replication SEs.

^h^ Any smokeless tobacco use was defined as smokeless and/or snus use.

**Table 2.** Association Between First Tobacco Product Flavored Among Young Adult Ever Tobacco Users at Wave 1 and Product-Specific Tobacco Use at Wave 2 of the Population Assessment of Tobacco and Health Study

Ever Tobacco Use at Wave 1	Past 12-mo Use at Wave 2^a^		Past 30-d Use at Wave 2^b^		≥6 d Use at Wave 2^c^		≥20 d Use at Wave 2^c^		Daily Use at Wave 2^d^		Current Regular Use at Wave 2^d^	
	Participants, No. (%)	aPR (95% CI)^e^	Participants, No. (%)	aPR (95% CI)^e^	Participants, No. (%)	aPR (95% CI)^e^	Participants, No. (%)	aPR (95% CI)^e^	Participants, No. (%)	aPR (95% CI)^e^	Participants, No. (%)	aPR (95% CI)^e^
Ever cigarette use												
First product nonflavored	1577 (63.2)	1 [Reference]	1248 (48.7)	1 [Reference]	879 (33.7)	1 [Reference]	719 (26.8)	1 [Reference]	631 (22.9)	1 [Reference]	929 (35.2)	1 [Reference]
First product flavored	1772 (69.3)	1.09 (1.04-1.15)	1458 (55.7)	1.13 (1.06-1.21)	1060 (40.1)	1.17 (1.07-1.29)	884 (33.1)	1.22 (1.10-1.36)	773 (28.6)	1.25 (1.11-1.41)	1077 (40.8)	1.17 (1.07-1.27)
First product menthol or mint flavored^f^	1635 (69.8)	1.10 (1.05-1.16)	1357 (56.5)	1.15 (1.07-1.23)	1005 (41.6)	1.21 (1.10-1.32)	841 (34.5)	1.26 (1.13-1.41)	742 (30)	1.29 (1.15-1.46)	1016 (41.7)	1.18 (1.09-1.29)
Ever e-cigarette use												
First product nonflavored	697 (61.3)	1 [Reference]	267 (24.1)	1 [Reference]	94 (8.6)	1 [Reference]	62 (5.9)	1 [Reference]	40 (3.7)	1 [Reference]	111 (9)	1 [Reference]
First product flavored	1324 (71.4)	1.17 (1.10-1.25)	571 (30.7)	1.31 (1.10-1.55)	238 (13.3)	1.67 (1.27-2.20)	163 (9.2)	1.67 (1.21-2.31)	141 (7.8)	2.30 (1.61-3.28)	330 (17.3)	2.05 (1.61-2.61)
Ever any cigar use												
First product nonflavored	703 (45)	1 [Reference]	445 (27.2)	1 [Reference]	82 (4.9)	1 [Reference]	46 (2.6)	1 [Reference]	33 (1.8)	1 [Reference]	128 (7.9)	1 [Reference]
First product flavored	1445 (52.8)	1.19 (1.10-1.29)	870 (30)	1.13 (1.01-1.27)	154 (5.4)	1.17 (0.87-1.57)	71 (2.3)	0.84 (0.56-1.25)	50 (1.5)	0.88 (0.52-1.50)	314 (11.4)	1.60 (1.26-2.02)
Ever use of traditional cigars												
First product nonflavored	440 (45.5)	1 [Reference]	195 (19.9)	1 [Reference]	NA^g^	1 [Reference]	NA^g^	NA^g^	NA^g^	NA^g^	57 (5.9)	1 [Reference]
First product flavored	319 (48.8)	1.15 (1.00-1.32)	126 (18)	0.96 (0.72-1.28)	17 (2)	NA^h^	NA^g^	NA^g^	NA^g^	NA^g^	56 (7.8)	1.39 (0.92-2.10)
Ever use of cigarillos												
First product nonflavored	553 (36.5)	1 [Reference]	354 (22.6)	1 [Reference]	54 (3.5)	1 [Reference]	25 (1.6)	1 [Reference]	18 (1.1)	1 [Reference]	87 (6.1)	1 [Reference]
First product flavored	1067 (43.3)	1.22 (1.11-1.35)	631 (24.2)	1.13 (0.98-1.31)	89 (3.4)	1.02 (0.69-1.51)	33 (1.1)	NA^h^	24 (0.7)	NA^h^	201 (7.9)	1.49 (1.08-2.05)
Ever use of filtered cigars												
First product nonflavored	146 (22.4)	1 [Reference]	70 (10.1)	1 [Reference]	NA^g^	1 [Reference]	NA^g^	NA^g^	NA^g^	NA^g^	18 (2.3)	1 [Reference]
First product flavored	314 (32.1)	1.50 (1.25-1.81)	138 (12.8)	1.32 (0.97-1.78)	27 (2.5)	NA^h^	NA^g^	NA^g^	NA^g^	NA^g^	68 (6.8)	3.69 (2.08-6.57)
Ever use of pipe tobacco												
First product nonflavored	168 (18.7)	1 [Reference]	72 (7.8)	1 [Reference]	NA^g^	NA^g^	NA^g^	NA^g^	NA^g^	NA^g^	32 (3.9)	1 [Reference]
First product flavored	72 (21.4)	1.15 (0.86-1.55)	34 (9.9)	1.25 (0.76-2.06)	NA^g^	NA^g^	NA^g^	NA^g^	NA^g^	NA^g^	25 (8.2)	2.28 (1.23-4.24)
Ever use of hookah tobacco												
First product nonflavored	186 (39.1)	1 [Reference]	89 (18.8)	1 [Reference]	NA^g^	1 [Reference]	NA^g^	1 [Reference]	NA^g^	1 [Reference]	30 (6.4)	1 [Reference]
First product flavored	1868 (51.5)	1.33 (1.14-1.54)	783 (21.3)	1.26 (0.99-1.61)	116 (3)	2.36 (1.00-5.54)	43 (1)	NA^h^	27 (0.6)	NA^h^	451 (12.6)	1.91 (1.23-2.98)
Ever use of any smokeless tobacco^i^												
First product nonflavored	122 (31.1)	1 [Reference]	91 (23.4)	1 [Reference]	49 (12.8)	1 [Reference]	40 (10.9)	1 [Reference]	37 (9.9)	1 [Reference]	62 (15.6)	1 [Reference]
First product flavored	509 (44.5)	1.40 (1.16-1.69)	360 (30.7)	1.21 (0.96-1.54)	220 (19.5)	NA^h^	171 (15)	NA^h^	144 (12.3)	NA^h^	290 (24.7)	1.54 (1.08-2.20)
Ever use of smokeless tobacco (excluding snus)												
First product nonflavored	158 (37.3)	1 [Reference]	123 (29.2)	1 [Reference]	77 (18.8)	1 [Reference]	57 (14.1)	1 [Reference]	55 (12.9)	1 [Reference]	92 (21.9)	1 [Reference]
First product flavored	387 (47.2)	1.29 (1.09-1.51)	289 (34.2)	1.18 (0.96-1.46)	176 (21.2)	NA^h^	144 (17.3)	NA^h^	120 (14.4)	NA^h^	231 (26.8)	1.28 (0.99-1.66)
Ever use of snus												
First product nonflavored	38 (18.4)	1 [Reference]	20 (9.1)	1 [Reference]	NA^g^	1 [Reference]	NA^g^	NA^g^	NA^g^	NA^g^	NA^g^	1 [Reference]
First product flavored	201 (28.2)	1.61 (1.13-2.31)	85 (11.3)	NA^h^	23 (3.1)	NA^h^	NA^g^	NA^g^	NA^g^	NA^g^	50 (7.1)	NA^h^
Ever use of dissolvable tobacco												
First product nonflavored	NA^g^	NA^g^	NA^g^	NA^g^	NA^g^	NA^g^	NA^g^	NA^g^	NA^g^	NA^g^	NA^g^	NA^g^
First product flavored	NA^g^	NA^g^	NA^g^	NA^g^	NA^g^	NA^g^	NA^g^	NA^g^	NA^g^	NA^g^	NA^g^	NA^g^

Abbreviations: aPR, adjusted prevalence ratio; NA, not applicable.

^a^ Past 12-month use was defined as smoked or used product (even 1 or 2 times) in the past 12 months. Individuals who responded “don’t know” or refused to answer were excluded from the denominator. Unweighted numbers (percentages) of respondents excluded from the denominator for past 12-month use are as follows: cigarettes, 1 (0.0%); e-cigarettes, 110 (3.5%); any cigar, 3 (0.1%); traditional cigars, 0 (0.0%); cigarillos, 1 (0.0%); filtered cigars, 2 (0.1%); pipe, 1 (0.1%); hookah, 2 (0.1%); any smokeless product, 4 (0.3%); smokeless product, 1 (0.1%); and snus, 2 (0.2%).

^b^ Past 30-day use was defined as smoked or used product (even 1 or 2 times) in the past 30 days. Individuals who responded “don’t know” or refused to answer were excluded from the denominator. Unweighted numbers (percentages) of respondents excluded from the denominator for past 30-day use were as follows: cigarettes, 0 (0.0%); e-cigarettes, 111 (3.6%); any cigar, 2 (0.1%); traditional cigars, 0 (0.0%); cigarillos, 4 (0.1%); filtered cigars, 1 (0.1%); pipe, 1 (0.1%); hookah, 2 (0.1%); any smokeless product, 3 (0.2%); smokeless product, 3 (0.2%); and snus, 1 (0.1%).

^c^ Moderate use was defined as having smoked or used the product on at least 6 of the past 30 days. Frequent product use defined as having smoked or used the product on at least 20 of the past 30 days. Individuals who responded “don’t know” or refused to answer were excluded from the denominator. Unweighted numbers (percentages) of respondents excluded from the denominator for moderate and frequent use were as follows: cigarettes moderate or frequent use, 81 (1.7%); e-cigarettes moderate or frequent use, 181 (5.8%); any cigar product moderate use, 305 (7.5%); any cigar product frequent use, 315 (7.7%); traditional cigars moderate use, 126 (7.9%); cigarillos moderate or frequent use, 152 (4.1%); filtered cigars moderate use, 60 (3.9%); hookah moderate or frequent use, 8 (0.2%); any smokeless tobacco product moderate use, 60 (4.0%); any smokeless tobacco product frequent use, 66 (4.4%); smokeless tobacco moderate or frequent use, 40 (3.3%); and snus moderate use, 28 (3.1%).

^d^ Daily use among adults was defined as now smokes or uses product every day. Current regular use was defined for cigarettes as having smoked at least 100 cigarettes in lifetime and now smokes every day or some days; for all other products, regular use was defined as having ever used a product “fairly regularly” and now uses it every day or some days. Individuals who responded “don’t know” or refused to answer were excluded from the denominator. Unweighted numbers (percentages) of respondents excluded from the denominator for daily and current regular use were as follows: cigarettes daily use, 30 (0.6%); cigarettes current established use, 3 (0.1%); e-cigarettes daily use, 71 (2.3%); e-cigarettes current established use, 10 (0.3%); any cigar product daily use, 116 (2.9%); any cigar product current established use, 247 (6.1%); traditional cigars current established use, 0 (0.0%); cigarillos daily use, 76, (2.1%); cigarillos current established use, 270 (7.4%); filtered cigars current established use, 1 (0.1%); pipe tobacco current established use, 0 (0.0%); hookah daily use, 5 (0.1%); hookah current established use, 0 (0.0%); any smokeless tobacco product daily use, 45 (3.0%); any smokeless tobacco product current established use, 6 (0.4%); smokeless tobacco daily use, 28 (2.3%); smokeless tobacco current established use, 0 (0.0%); and snus current established use, 0 (0.0%).

^e^ Multivariable modified Poisson regression models among young adults were adjusted for age, sex, race/ethnicity, education, income, past 30-day alcohol, marijuana, or other drug use, and 3 Global Appraisal of Individual Needs–Short Screener subscales (internalizing problems, externalizing problems, and substance use problems). Individuals who reported “don’t know” or refused to answer any of these items were treated as missing. Among young adults, data were missing for age (0 participants [0.0%]), sex (0 participants [0.0%]), race/ethnicity (0 participants [0.0%]), education (0 participants [0.0%]), income (589 participants [10.1%]), past 30-day alcohol, marijuana, or other drug use (54 participants [0.9%]), internalizing problems subscale (49 participants [0.8%]), externalizing problems subscale (75 participants [1.3%]), and substance use problems subscale (101 participants [1.7%]). Respondents who reported never alcohol, marijuana, or other drug use were categorized as non–past 30-day users.

^f^ A separate comparison was made between wave 1 cigarette smokers who first smoked a menthol or mint flavored cigarette vs smokers who first smoked a nonflavored cigarette. Individuals who reported first other flavored cigarette use were excluded from the denominator.

^g^ The estimate was suppressed because it has low statistical precision. It is based on a sample size of less than 50, or the coefficient of variation of the estimate is larger than 30%.

^h^ There was insufficient observations to compute balanced repeated replication SEs.

^i^ Any smokeless tobacco use was defined as smokeless and/or snus use.

**Table 3.** Association Between First Tobacco Product Flavored Among Adults Aged 25 Years and Older Ever Tobacco Users at Wave 1 and Product-Specific Tobacco Use at Wave 2 of the Population Assessment of Tobacco and Health Study

Ever Tobacco Use at Wave 1	Past 12-mo Use at Wave 2^a^		Past 30-d Use at Wave 2^b^		≥6 d Use at Wave 2^c^		≥20 day Use at Wave 2^c^		Daily Use at Wave 2^d^		Current Regular Use at Wave 2^d^	
	Participants, No. (%)	aPR (95%CI)^e^	Participants, No. (%)	aPR (95%CI)^e^	Participants, No. (%)	aPR (95% CI)^e^	Participants, No. (%)	aPR (95% CI)^e^	Participants, No. (%)	aPR (95% CI)^e^	Participants, No. (%)	aPR (95% CI)^e^
Ever cigarette use												
First product nonflavored	5499 (31.5)	1 [Reference]	4946 (27.8)	1 [Reference]	4374 (24)	1 [Reference]	4028 (21.9)	1 [Reference]	3721 (20)	1 [Reference]	4430 (24.4)	1 [Reference]
First product flavored	3740 (41.9)	1.10 (1.05-1.15)	3371 (37.1)	1.09 (1.04-1.14)	2963 (31.9)	1.08 (1.03-1.14)	2688 (28.7)	1.08 (1.02-1.14)	2447 (26)	1.09 (1.03-1.16)	2986 (32.3)	1.11 (1.06-1.17)
First product menthol or mint flavored^f^	3554 (42.9)	1.13 (1.08-1.18)	3218 (38.1)	1.12 (1.07-1.17)	2846 (32.9)	1.12 (1.06-1.17)	2590 (29.8)	1.12 (1.06-1.18)	2367 (27.1)	1.13 (1.07-1.20)	2867 (33.4)	1.14 (1.09-1.20)
Ever e-cigarette use												
First product nonflavored	1793 (57.7)	1 [Reference]	801 (25.1)	1 [Reference]	356 (10.8)	1 [Reference]	250 (7.6)	1 [Reference]	217 (6.3)	1 [Reference]	441 (13)	1 [Reference]
First product flavored	1818 (66.5)	1.15 (1.10-1.20)	838 (30.8)	1.25 (1.13-1.38)	440 (16.3)	1.64 (1.42-1.89)	338 (12.8)	1.85 (1.54-2.22)	299 (11)	1.87 (1.55-2.26)	580 (19.9)	1.60 (1.41-1.82)
Ever any cigar use												
First product nonflavored	1613 (20.1)	1 [Reference]	942 (11.1)	1 [Reference]	256 (2.8)	1 [Reference]	167 (1.8)	1 [Reference]	134 (1.4)	1 [Reference]	445 (4.9)	1 [Reference]
First product flavored	1636 (30.1)	1.31 (1.22-1.41)	1016 (18.1)	1.34 (1.22-1.48)	272 (4.7)	1.36 (1.08-1.70)	175 (3.1)	1.44 (1.06-1.95)	137 (2.3)	1.49 (1.08-2.07)	491 (8.4)	1.56 (1.29-1.87)
Ever use of traditional cigars												
First product nonflavored	1386 (19.6)	1 [Reference]	632 (8.7)	1 [Reference]	84 (1)	1 [Reference]	39 (0.5)	1 [Reference]	33 (0.4)	1 [Reference]	287 (3.7)	1 [Reference]
First product flavored	476 (23.2)	1.09 (0.97-1.23)	264 (12.8)	1.36 (1.15-1.62)	38 (1.6)	1.15 (0.70-1.88)	19 (0.8)	1.37 (0.69-2.74)	18 (0.7)	1.71 (0.84-3.49)	109 (4.7)	1.44 (1.10-1.87)
Ever use of cigarillos												
First product nonflavored	762 (12.6)	1 [Reference]	474 (7.8)	1 [Reference]	115 (1.9)	1 [Reference]	74 (1.2)	1 [Reference]	60 (1)	1 [Reference]	189 (3.1)	1 [Reference]
First product flavored	891 (20.6)	1.37 (1.22-1.54)	553 (12.4)	1.23 (1.05-1.44)	112 (2.5)	1.02 (0.75-1.39)	57 (1.3)	0.88 (0.58-1.35)	37 (0.8)	0.65 (0.42-1.00)	211 (4.6)	1.29 (1.01-1.64)
Ever use of filtered cigars												
First product nonflavored	362 (11.5)	1 [Reference]	202 (6.4)	1 [Reference]	95 (3)	1 [Reference]	80 (2.6)	1 [Reference]	70 (2.3)	1 [Reference]	118 (3.7)	1 [Reference]
First product flavored	373 (18.2)	1.55 (1.31-1.83)	203 (10)	1.50 (1.16-1.95)	87 (4.1)	1.70 (1.16-2.50)	65 (3.1)	1.53 (0.98-2.38)	58 (2.8)	1.65 (1.03-2.63)	131 (6.3)	1.79 (1.25-2.54)
Ever use of pipe tobacco												
First product nonflavored	240 (5.3)	1 [Reference]	136 (3.1)	1 [Reference]	43 (0.8)	1 [Reference]	34 (0.6)	1 [Reference]	30 (0.6)	1 [Reference]	92 (1.7)	1 [Reference]
First product flavored	146 (7.3)	1.42 (1.07-1.87)	72 (3.6)	1.24 (0.86-1.78)	NA^g^	NA^g^	NA^g^	NA^g^	NA^g^	NA^g^	47 (2.4)	1.44 (0.92-2.25)
Ever use of hookah tobacco												
First product nonflavored	127 (10.6)	1 [Reference]	57 (4.6)	1 [Reference]	NA^g^	1 [Reference]	NA^g^	NA^g^	NA^g^	NA^g^	NA^g^	1 [Reference]
First product flavored	738 (20.7)	1.63 (1.25-2.13)	264 (7.3)	1.28 (0.88-1.87)	34 (0.9)	NA^h^	NA^g^	NA^g^	NA^g^	NA^g^	136 (3.7)	5.66 (2.04-15.71)
Ever use of any smokeless tobacco^i^												
First product nonflavored	453 (15.9)	1 [Reference]	362 (12.9)	1 [Reference]	298 (10.4)	1 [Reference]	261 (9)	1 [Reference]	238 (8.2)	1 [Reference]	318 (11.2)	1 [Reference]
First product flavored	802 (26.1)	1.50 (1.32-1.70)	622 (20.4)	1.49 (1.28-1.73)	472 (15.3)	1.43 (1.20-1.70)	409 (13.3)	1.44 (1.17-1.78)	369 (11.8)	1.44 (1.15-1.80)	564 (18.1)	1.55 (1.32-1.82)
Ever use of smokeless tobacco (excluding snus)												
First product nonflavored	487 (16.5)	1 [Reference]	402 (13.7)	1 [Reference]	329 (11.2)	1 [Reference]	292 (9.8)	1 [Reference]	264 (8.8)	1 [Reference]	360 (12.2)	1 [Reference]
First product flavored	632 (24.8)	1.38 (1.21-1.58)	523 (20.6)	1.43 (1.22-1.67)	403 (15.5)	1.37 (1.16-1.62)	351 (13.5)	1.36 (1.12-1.67)	317 (12)	1.38 (1.10-1.72)	471 (18.1)	1.43 (1.21-1.68)
Ever use of snus												
First product nonflavored	53 (10.8)	1 [Reference]	25 (5.1)	1 [Reference]	NA^g^	1 [Reference]	NA^g^	1 [Reference]	NA^g^	1 [Reference]	NA^g^	1 [Reference]
First product flavored	209 (19.4)	1.71 (1.20-2.44)	102 (9.5)	1.60 (0.95-2.71)	47 (4.2)	1.82 (0.81-4.09)	34 (3.1)	2.50 (0.80-7.76)	31 (2.9)	2.17 (0.71-6.59)	75 (6.8)	3.20 (1.65-6.20)
Ever use of dissolvable tobacco												
First product nonflavored	NA^g^	NA^g^	NA^g^	NA^g^	NA^g^	NA^g^	NA^g^	NA^g^	NA^g^	NA^g^	NA^g^	NA^g^
First product flavored	NA^g^	NA^g^	NA^g^	NA^g^	NA^g^	NA^g^	NA^g^	NA^g^	NA^g^	NA^g^	NA^g^	NA^g^

Abbreviations: aPR, adjusted prevalence ratio; NA, not applicable.

^a^ Past 12-month use was defined as smoked or used product (even 1 or 2 times) in the past 12 months. Individuals who responded “don’t know” or refused to answer were excluded from the denominator. Unweighted numbers (percentages) of respondents excluded from the denominator for past 12-month use were as follows: cigarettes, 4 (0.0%); e-cigarettes, 448 (7.2%); any cigar, 7 (0.1%); traditional cigars, 5 (0.1%); cigarillos, 3 (0.0%); filtered cigars, 3 (0.1%); pipe, 5 (0.1%); hookah, 1 (0.0%); any smokeless product, 4 (0.1%); smokeless product, 1 (0.0%); and snus, 1 (0.1%).

^b^ Past 30-day use was defined as smoked or used product (even 1 or 2 times) in the past 30 days. Individuals who responded “don’t know” or refused to answer were excluded from the denominator. Unweighted numbers (percentages) of respondents excluded from the denominator for past 30-day use were as follows: cigarettes, 5 (0.0%); e-cigarettes, 455 (7.3%); any cigar, 8 (0.1%); traditional cigars, 5 (0.1%); cigarillos, 9 (0.1%); filtered cigars, 4 (0.1%); pipe, 9 (0.2%); hookah, 1 (0.0%); any smokeless product, 4 (0.1%); smokeless product, 2 (0.0%); and snus, 2 (0.1%).

^c^ Moderate use was defined as having smoked or used the product on at least 6 of the past 30 days. Frequent product use was defined as having smoked or used the product on at least 20 of the past 30 days. Individuals who responded “don’t know” or refused to answer were excluded from the denominator. Unweighted numbers (percentages) of respondents excluded from the denominator for moderate and frequent use were as follows: cigarettes moderate or frequent use, 117 (0.7%); e-cigarettes moderate or frequent use, 326 (5.2%); any cigar product moderate use, 623 (6.2%); any cigar product frequent use, 638 (6.3%); traditional cigars moderate or frequent use, 388 (5.7%); cigarillos moderate or frequent use, 221 (2.9%); filtered cigars moderate or frequent use, 76 (1.9%); pipe tobacco moderate or frequent use, 84 (1.8%); hookah moderate use, 2 (0.0%); any smokeless product moderate use, 88 (2.0%); any smokeless product frequent use, 90 (2.0%); smokeless tobacco moderate or frequent use, 64 (1.6%); snus moderate or frequent use, 34 (2.4%).

^d^ Daily use among adults was defined as now smokes or uses product every day. Current regular use was defined for cigarettes as having smoked at least 100 cigarettes in lifetime and now smokes every day or some days; for all other products, current regular use was defined as having ever used product “fairly regularly” and now uses it every day or some days. Individuals who responded “don’t know” or refused to answer were excluded from the denominator. Unweighted numbers (percentages) of respondents excluded from the denominator for daily and current regular use were as follows: cigarettes daily use, 92 (0.6%); cigarettes current established use, 25 (0.2%); e-cigarettes daily use, 134 (2.2%); e-cigarettes current established use, 31 (0.5%); any cigar product daily use, 220 (2.2%); any cigar product current established use, 98 (1.0%); traditional cigars daily use, 112 (1.7%); traditional cigars current established use, 2 (0.0%); cigarillos daily use, 90 (1.2%); cigarillos current established use, 101 (1.3%); filtered cigars daily use, 29 (0.7%); filtered cigars current established use, 3 (0.1%); pipe tobacco daily use, 49 (1.0%); pipe tobacco current established use, 3 (0.1%); hookah current established use, 0 (0.0%); any smokeless product daily use, 56 (1.3%); any smokeless product current established use, 8 (0.2%); smokeless tobacco daily use, 38 (1.0%); smokeless tobacco current established use, 4 (0.1%); snus daily use, 20, (1.4%); and snus current established use, 1 (0.1%).

^e^ Multivariable modified Poisson regression models among adults were adjusted for age, sex, race/ethnicity, education, income, past 30-day alcohol, marijuana, or other drug use, and 3 Global Appraisal of Individual Needs–Short Screener subscales (internalizing problems, externalizing problems, and substance use problems). Individuals who reported “don’t know” or refused to answer any of these items were treated as missing. Among adults, data were missing for age (0 participants [0.0%]), sex (0 participants [0.0%]), race/ethnicity (0 participants [0.0%]), education (0 participants [0.0%]), income (1263 participants [7.6%]), past 30-day alcohol, marijuana, or other drug use (247 participants [1.5%]), internalizing problems subscale (199 participants [1.2%]), externalizing problems subscale (376 participants [2.3%]), and substance use problems subscale (427 participants [2.6%]). Respondents who reported never alcohol, marijuana, or other drug use were categorized as non–past 30-day users.

^f^ A separate comparison was made between wave 1 cigarette smokers who first smoked a menthol or mint flavored cigarette vs smokers who first smoked a nonflavored cigarette. Individuals who reported first other flavored cigarette use were excluded from the denominator.

^g^ The estimate was suppressed because it has low statistical precision. It is based on a sample size of less than 50, or the coefficient of variation of the estimate is larger than 30%.

^h^ There were insufficient observations to compute balanced repeated replication standard errors.

^i^ Any smokeless tobacco use was defined as smokeless and/or snus use.

Among young adults, first use of flavored e-cigarettes (aPR, 2.05; 95% CI, 1.61-2.61), any cigars (aPR, 1.60; 95% CI, 1.26-2.02), cigarillos (aPR, 1.49; 95% CI, 1.08-2.05), filtered cigars (aPR, 3.69; 95% CI, 2.08-6.57), hookah (aPR, 1.91; 95% CI, 1.23-2.98), and any smokeless tobacco (aPR, 1.54; 95% CI, 1.08-2.20) was prospectively associated with current regular use of those products at wave 2 compared with first nonflavored use (Table 2). Among adults aged 25 years and older, first use of flavored e-cigarettes (aPR, 1.60; 95% CI, 1.41-1.82), any cigars (aPR, 1.56; 95% CI, 1.29-1.87), cigarillos (aPR, 1.29; 95% CI, 1.01-1.64), filtered cigars (aPR, 1.79; 95% CI, 1.25-2.54), hookah (aPR, 5.66; 95% CI, 2.04-15.71), and any smokeless tobacco (aPR, 1.55; 95% CI, 1.32-1.82) was prospectively associated with current regular use of those products at wave 2 compared with first nonflavored use (Table 3). First use of any flavored cigar product was positively associated with each measure of subsequent use among adults aged 25 years and older (12-month use aPR, 1.31 [95% CI, 1.22-1.41]; 30-day use aPR, 1.34 [95% CI, 1.22-1.48]; ≥6-day use aPR, 1.36 [95% CI, 1.08-1.70]; ≥20-day use aPR, 1.44 [95% CI, 1.06-1.95]; daily use aPR, 1.49 [95% CI, 1.08-2.07]; current regular use aPR, 1.56 [95% CI, 1.29-1.87]) (Table 3) and with past 12-month (aPR, 1.19; 95% CI, 1.10-1.29), past 30-day (aPR, 1.13; 95% CI, 1.01-1.27), and current regular use (aPR, 1.60; 95% CI, 1.26-2.02) among young adults (Table 2).

### Association Between First Use of a Given Tobacco Product Being Flavored at Wave 1 and Frequency of Current Tobacco Use at Wave 2

Among both youth and adults aged 25 years and older, first flavored or menthol cigarette use assessed at wave 1 was associated with a higher relative risk of cigarette use in the past 12 months (youth, flavored, relative risk ratio [RRR], 1.47 [95% CI, 1.09-1.98] and menthol, RRR, 1.60 [95% CI, 1.17-2.21]; adults, flavored, RRR, 1.34 [95% CI, 1.09-1.63] and menthol, RRR, 1.40 [95% CI, 1.14-1.73]), on 1 to 5 of the past 30 days (youth, flavored, RRR, 1.69 [95% CI, 1.20-2.40] and menthol, RRR, 1.93 [95% CI, 1.32-2.83]; adults, flavored RRR, 1.30 [95% CI, 1.07-1.58] and menthol, RRR, 1.36 [95% CI, 1.10-1.67]), and on all 30 of the past 30 days (youth, flavored, RRR, 1.61 [95% CI, 1.10-2.38] and menthol, RRR, 1.88 [95% CI, 1.25-2.82]; adults, flavored, RRR, 1.23 [95% CI, 1.11-1.35] and menthol, RRR, 1.32 [95% CI, 1.20-1.45]) compared with nonflavored cigarette use (Table 4). Among young adults, first flavored or menthol cigarette use was associated with use on all 30 days (RRR, 1.56 [95% CI, 1.27-1.93] for flavored and 1.66 [95% CI, 1.33-2.06] for menthol). Among young adults, first flavored e-cigarette use at wave 1 was associated with higher relative risk of e-cigarette use in the past 12 months (RRR, 1.52; 95% CI, 1.21-1.92), on 1 to 5 of the past 30 days (RRR, 1.61; 95% CI, 1.24-2.10), on 6 to 19 of the past 30 days (RRR, 2.35; 95% CI, 1.27-4.34), and on all 30 of the past 30 days (RRR, 3.24; 95% CI, 2.16-4.86) compared with nonflavored e-cigarette use. Among adults aged 25 years and older, first use of a flavored e-cigarette assessed at wave 1 was associated with greater frequency of e-cigarette use at wave 2 across all categories (past 12-month use, RRR, 1.38 [95% CI, 1.19-1.61]; 1-5 days in the past 30 days, RRR, 1.25 [95% CI, 1.02-1.53]; 6-19 days in the past 30 days, RRR, 1.44 [95% CI, 1.03-2.01]; 20-29 days in the past 30 days, RRR, 2.09 [95% CI, 1.09-4.00]; and all 30 of the past 30 days, RRR, 2.38 [95% CI, 1.90-3.00]).

**Table 4.** Multivariable Multinomial Logistic Regression Models of Frequency of Use at Wave 2 Among Ever Users of Specified Product at Wave 1 of the Population Assessment of Tobacco and Health Study, by Age Group

Age Group	Participants, No.	No Past 12-mo Use	RRR (95% CI)				
			Past 12-mo Use, No Past 30-d Use	1-5 d in the Past 30 d	6-19 d in the Past 30 d	20-29 d in the Past 30 d	All 30 d in the Past 30 d
Youth^a^							
First cigarette flavored	1316	1 [Reference]	1.47 (1.09-1.98)	1.69 (1.20-2.40)	1.22 (0.72-2.07)	1.15 (0.61-2.18)	1.61 (1.10-2.38)
First cigarette menthol or mint flavored^b^	1223	1 [Reference]	1.60 (1.17-2.21)	1.93 (1.32-2.83)	1.33 (0.77-2.31)	1.23 (0.65-2.32)	1.88 (1.25-2.82)
First e-cigarette flavored	1045	1 [Reference]	1.26 (0.82-1.94)	1.30 (0.78-2.16)	1.40 (0.64-3.07)	1.08 (0.21-5.71)	2.85 (0.94-8.63)
Young adults^c^							
First cigarette flavored	4109	1 [Reference]	1.13 (0.90-1.41)	1.24 (1.00-1.55)	1.21 (0.93-1.57)	1.26 (0.86-1.86)	1.56 (1.27-1.93)
First cigarette menthol or mint flavored^b^	3925	1 [Reference]	1.13 (0.89-1.44)	1.21 (0.96-1.52)	1.24 (0.95-1.63)	1.30 (0.87-1.95)	1.66 (1.33-2.06)
First e-cigarette flavored	2622	1 [Reference]	1.52 (1.21-1.92)	1.61 (1.24-2.10)	2.35 (1.27-4.34)	0.81 (0.37-1.75)	3.24 (2.16-4.86)
Adults^d^							
First cigarette flavored	13 959	1 [Reference]	1.34 (1.09-1.63)	1.30 (1.07-1.58)	1.22 (0.96-1.56)	1.11 (0.86-1.43)	1.23 (1.11-1.35)
First cigarette menthol or mint flavored^b^	13 594	1 [Reference]	1.40 (1.14-1.73)	1.36 (1.10-1.67)	1.28 (1.00-1.63)	1.15 (0.89-1.48)	1.32 (1.20-1.45)
First e-cigarette flavored	5188	1 [Reference]	1.38 (1.19-1.61)	1.25 (1.02-1.53)	1.44 (1.03-2.01)	2.09 (1.09-4.00)	2.38 (1.90-3.00)

Abbreviation: RRR, relative risk ratio.

^a^ Multivariable multinomial logistic regression models among youth were adjusted for sex, race/ethnicity, education, past 30-day alcohol, marijuana, or other drug use, and 3 Global Appraisal of Individual Needs–Short Screener (GAIN-SS) subscales (internalizing problems, externalizing problems, and substance use problems). Individuals who reported “don’t know” or refused to answer any of these items were treated as missing. Among youth ever tobacco users, data were missing for sex (0 participants [0.0%]), race/ethnicity (27 participants [1.5%]), education (114 participants [6.2%]), past 30-day alcohol, marijuana, or other drug use (47 participants [2.6%]), internalizing problems subscale (40 participants [2.2%]), externalizing problems subscale (67 participants [3.7%]), and substance use problems subscale (71 participants [3.9%]). Respondents who reported never alcohol, marijuana, or other drug use were categorized as non–past 30-day users.

^b^ A separate comparison was made between wave 1 cigarette smokers who first smoked a menthol or mint flavored cigarette vs smokers who first smoked a nonflavored cigarette. Individuals who reported first other flavored cigarette use were excluded from the denominator.

^c^ Multivariable multinomial logistic regression models among young adults were adjusted for age, sex, race/ethnicity, education, income, past 30-day alcohol, marijuana, or other drug use, and 3 GAIN-SS subscales (internalizing problems, externalizing problems, and substance use problems). Individuals who reported “don't know” or refused to answer any of these items were treated as missing. Among young adults, data were missing for age (0 participants [0.0%]), sex (0 participants [0.0%]), race/ethnicity (0 participants [0.0%]), education (0 participants [0.0%]), income (449 participants [9.5%]), past 30-day alcohol, marijuana, or other drug use (46 participants [1.0%]), internalizing problems subscale (42 participants [0.9%]), externalizing problems subscale (60 participants [1.3%]), and substance use problems subscale (83 participants [1.8%]). Respondents who reported never alcohol, marijuana, or other drug use were categorized as non–past 30-day users.

^d^ Multivariable multinomial logistic regression models among adults were adjusted for age, sex, race/ethnicity, education, income, past 30-day alcohol, marijuana, or other drug use, and 3 GAIN-SS subscales (internalizing problems, externalizing problems, and substance use problems). Individuals who reported “don’t know” or refused to answer any of these items were treated as missing. Among adults, data were missing for age (0 participants [0.0%]), sex (0 participants [0.0%]), race/ethnicity (0 participants [0.0%]), education (0 participants [0.0%]), income (1216 participants [7.6%]), past 30-day alcohol, marijuana, or other drug use (234 participants [1.5%]), internalizing problems subscale (185 participants [1.2%]), externalizing problems subscale (359 participants [2.3%]), and substance use problems subscale (411 participants [2.6%]). Respondents who reported never alcohol, marijuana, or other drug use were categorized as non–past 30-day users.

## Discussion

The current study found that (1) youth and young adults who were new users of a tobacco product at wave 2 (over the 10- to 13-month follow-up period) were more likely to try flavored tobacco products than adults; (2) first use of a flavored cigarette documented at wave 1 was positively associated with past 12-month and past 30-day cigarette use among youth, young adults, and adults aged 25 years and older at wave 2; (3) first use of a menthol or mint flavored cigarette documented at wave 1 was positively associated with past 12-month and past 30-day cigarette use at wave 2 in all age groups; (4) first use of flavored e-cigarettes, cigars, hookah, and smokeless tobacco was associated with subsequent use of those products at wave 2 among young adults and adults aged 25 years and older; (5) first flavored use of a cigarette, e-cigarette, any cigar, cigarillo, filtered cigar, hookah, and any smokeless tobacco documented at wave 1 was associated with current regular use of those products among young adults and adults aged 25 years and older at wave 2 compared with first use of a nonflavored product; (6) first flavored or menthol cigarette use was associated with progression to daily cigarette use at wave 2 in all age groups; and (7) first flavored e-cigarette use was associated with progression of e-cigarette frequency among young adults and adults aged 25 years and older. The age gradient in first use of a flavored tobacco product and positive association with subsequent tobacco use are consistent with the findings from prior studies.^9,10,12,13,14,15,16,17,18^ These data support tobacco industry research^2,3,4,5^ on the role of flavors to promote uptake in nonusers. The observation of significant prospective associations between first use of a menthol or mint flavored cigarette at wave 1 and continued cigarette use at wave 2 across all age groups adds novel insight to previous longitudinal studies^28^ on the role of menthol in facilitating smoking initiation and progression and reducing cessation.

### Limitations

This study has several limitations. First, tobacco product use and flavored tobacco use in the questionnaire are based on the respondent’s perception of and ability to recall whether past or current products were flavored. Second, analyses examined continued use or progression of use over the 10- to 13-month follow-up period; thus, the analyses excluded participants who were missing data at 1 of the waves. The extent of missing data and the small number of observations for specific products limited the detection of certain associations from wave 1 to wave 2; this was especially an issue for the youth findings. Third, progression of tobacco use is known to occur over several years among young people,^29^ and flavored use among adults was asked only of established tobacco users at wave 1; future studies that include experimental tobacco users and a longer follow-up period will inform estimates of the association of flavored tobacco with uptake and maintenance of tobacco use. Fourth, analyses were stratified by age, and, among adults, age was also included as a covariate; this does not fully account for potential cohort effects given differences in the availability of flavored tobacco products at the time of initiation, or the fact that estimates for the group of adults aged 25 years and older may be inflated because of greater smoking cessation among older adults.

## Conclusions

The findings of this study suggest that flavors in most tobacco products are associated with youth and young adult tobacco experimentation; that first use of a menthol or mint flavored cigarette places youth, young adults, and adults aged 25 years and older at risk of subsequent cigarette smoking; and that first use of flavored e-cigarettes, cigars, hookah, and smokeless tobacco products can place young adults and adults at risk of regular tobacco use when examined prospectively. Additional longitudinal studies will allow for a better understanding of the role of flavors in tobacco use progression and trajectories over time.

## Notes

Supplement. **eTable.** Sociodemographic and Tobacco Use Constructs in the PATH Study
