# Supplementary material for: Association of Flavored Tobacco Use With Tobacco Initiation and Subsequent Use Among US Youth and Adults, 2013-2015
Source: JAMA Netw Open. 2019 Oct 23;2(10):e1913804. doi: 10.1001/jamanetworkopen.2019.13804 (PMC6820032; doi:10.1001/jamanetworkopen.2019.13804)
Supplement: Supplement. — eTable. Sociodemographic and Tobacco Use Constructs in the PATH Study [file jamanetwopen-2-e1913804-s001.pdf]

Supplementary Online Content

Villanti AC, Johnson AL, Glasser AM, et al. Association of flavored tobacco use with tobacco initiation and subsequent use among US youth and adults, 2013-2015. *JAMA Netw Open*. 2019;2(10):e1913804. doi:10.1001/jamanetworkopen.2019.13804

eTable. Sociodemographic and Tobacco Use Constructs in the PATH Study

This supplementary material has been provided by the authors to give readers additional information about their work.

© 2019 Villanti AC et al. *JAMA Network Open.*

eTable. Sociodemographic and Tobacco Use Constructs in the PATH Study

Construct	Definition
**Sociodemographics**	
Age	For young adults, self-reported age is dichotomized to ages 18-20 and 21-24 at Wave 1.<br>For adults 25+, self-reported age (continuous) at Wave 1.
Sex	Identifies as male or female at Wave 1.
Race/ethnicity	Categorized as non-Hispanic White, non-Hispanic Black, non-Hispanic Other, and Hispanic ethnicity at Wave 1.
Educational attainment	For youth, categorized as “less than high school”, “high school”, and “college or more” at Wave 1.<br>For adults, categorized as “less than high school”, “high school”, “some college or associate’s degree”, and “college or more” at Wave 1.
Annual household income	For adults, categorized as “less than $25,000”, “$25,000-$49,999”, “$50,000-$74,999”, and “$75,000 or more” at Wave 1.
Past 30-day use of alcohol	Consumed alcohol (even a sip) in the past 30 days at Wave 1.
Past 30-day use of marijuana	Smoked/used marijuana, hash, THC, grass, pot, or weed in the past 30 days at Wave 1.
Past 30-day use of other drugs	Used Ritalin, Adderall, painkillers, sedatives, tranquilizers, cocaine, crack, methamphetamines, speed, heroin, inhalants, solvents, or hallucinogens in the past 30 days at Wave 1.
Internalizing severity	Categorized as low (0-1 symptoms), moderate (2-3 symptoms), and high (4 symptoms) severity of internalizing disorder symptoms in the past year at Wave 1 using the Global Appraisal of Individual Needs - Short Screener (GAIN-SS).
Externalizing severity	Categorized as low (0-1 symptoms), moderate (2-3 symptoms), and high (4+ symptoms) severity of externalizing disorder symptoms in the past year at Wave 1 using the Global Appraisal of Individual Needs - Short Screener (GAIN-SS).
Substance use severity	Categorized as low (0-1 symptoms), moderate (2-3 symptoms), and high (4+ symptoms) severity of externalizing disorder symptoms in the past year at Wave 1 using the Global Appraisal of Individual Needs - Short Screener (GAIN-SS).
**Tobacco use**	
Ever use	Has ever smoked/used product, even one or two puffs/one or two times, at Wave 1.
New use	Started using a product between Wave 1 and Wave 2 (i.e., were never users at Wave 1 and past 12-month users at Wave 2)
Past 12-month use	Smoked/used product (even one or two puffs/one or two times) in the past 12 months at Wave 2.
Past 30-day use	Smoked/used product (even one or two puffs/one or two times) in the past 30 days at Wave 2.

© 2019 Villanti AC et al. *JAMA Network Open.*

Moderate use[i]	Smoked/used the product on at least 6 of the past 30 days at Wave 2.
Frequent use[ii]	Smoked/used the product on at least 20 of the past 30 days at Wave 2.
Daily use[iii]	For adults, now smokes/uses product every day at Wave 2.<br><br>For youth, smoked/used the product on 30 of the past 30 days at Wave 2.
Current regular use (adults only)	For cigarettes, has smoked at least 100 cigarettes in lifetime and now smokes every day or some days at Wave 2.<br><br>For all other products, has ever used product “fairly regularly” and now uses it every day or some days at Wave 2.
Frequency of use (mutually-exclusive categories)	An additional variable was created for use of each product to better capture frequency of tobacco use at Wave 2 among ever users at Wave 1; six mutually-exclusive categories were created:<br>• No past 12-month use;<br>• Past 12-month use, no past 30-day use;<br>• Use on 1-5 days in the past 30;<br>• Use on 6-19 days in the past 30;<br>• Use on 20-29 days in the past 30; and<br>• Use on all 30 days.
**Flavored tobacco product use**	
First use of a specific tobacco product was flavored	Cigarettes:<br>For adults, first started smoking cigarettes that tasted like menthol, mint, clove, spice, fruit, chocolate, alcoholic drinks, candy or other sweets (first use of any flavored cigarette); first started smoking cigarettes that tasted like menthol or mint (first use of a menthol/mint flavored cigarette; Wave 1, Wave 2).<br><br>For youth, first cigarette smoked was flavored to taste like menthol, mint, clove, spice, fruit, chocolate, alcoholic drinks, candy or other sweets (first use of any flavored cigarette); first cigarette smoked was flavored to taste like menthol or mint; Wave 1, Wave 2).<br><br>Non-cigarette products:<br>For adults, first started smoking/using [product] that tasted like menthol, mint, clove, spice, fruit, chocolate, alcoholic drinks, candy or other sweets (Wave 1, Wave 2).<br><br>For youth, first [product] smoked/used was flavored to taste like menthol, mint, clove, spice, fruit, chocolate, alcoholic drinks, candy or other sweets (Wave 1, Wave 2).

[i] For hookah, ‘moderate use’ was defined as smoking hookah 2 or more times per week or 6 or more times per month.

© 2019 Villanti AC et al. *JAMA Network Open.*

[ii] For hookah, 'frequent use' was having used hookah at least once in the past 30 days and using at least 20 times per month on average (which includes usually using every day, usually using weekly with use at least 5 times per week on average, and usually using monthly with use at least 20 times per month on average). Frequent use of 'any tobacco,' 'any cigar,' and 'smokeless including snus' reflects only those who used a particular product on 20 or more of the past 30 days.

[iii] For hookah, 'daily use' among youth was defined as smoking hookah 7 or more times per week or 30 or more times per month.

© 2019 Villanti AC et al. *JAMA Network Open.*
